# The surprise pathology—Primary squamous cell carcinoma of the colon—A case report

**DOI:** 10.1016/j.ijscr.2020.05.062

**Published:** 2020-05-30

**Authors:** Aysha Hassan Ali Husain, Kiran B. Kaundinya, Faizal Hammed, Abdul Rahim Al Sayed

**Affiliations:** Bahrain Defence Force Hospital, Bahrain

**Keywords:** Case report, Primary, Squamous cell carcinoma, Colon

## Abstract

**Introduction:**

Primary squamous cell carcinomas are rare in the colon. Identified as colonic growths causing obstructive or ulcerative features, they are treated like usual colonic adenocarcinomas until the surprise findings on histopathology. A thorough search for possible primary with colonic metastasis is warranted prior to confirmation of diagnosis. They can coexist with adenocarcinomas and ulcerative colitis. We present this case with intent to add to existing literature the presentation and catastrophic clinical course of the disease in our patient.

**Clinical findings:**

The patient presented with obstructive pattern of a colonic growth with rapid weight loss. There was no family history of colonic disease and the patient did not suffer from inflammatory bowel disease.

**Diagnosis and therapeutic intervention:**

CT Scan of the abdomen revealed the growth which was infiltrating the abdominal musculature causing micro abscess formation. Colonoscopy was inconclusive as the growth was not passable enough to obtain enough biopsy for pathology. The patient underwent surgery for removal of tumor and the histopathology revealed the squamous cell carcinoma. Through the course of patient’s recovery in hospital thorough evaluation was done to identify primary in sites mainly the urogenital tract. The patient was discharged and unfortunately succumbed to her disease at home before definitive treatment could be given.

**Conclusion:**

Squamous cell carcinomas in the colon warrants extensive search for the primary and coexistent adenocarcinomas or ulcerative colitis. In patients who recover from surgery, chemoradiation directed towards the pathology should be initiated to prevent rapid deterioration as in our case. Its presentation may be exophytic infiltrating surrounding structures and micro abscesses or perforations may also be encountered. We add our case report to the existing literature of primary squamous cell colon carcinoma series.

## Highlights

• Squamous cell carcinomas – primary is very rare in occurrence in the colorectal carcinoma group.
• They are either found independently or along with adenocarcinomas.
• They may be associated with metaplastic changes seen in inflammatory bowel disease or certain helminthiasis.
• They may have locoregional infiltration causing micro abscess or perforations.
• They are aggressive and may progress to deterioration early.

## 1 Introduction

Squamous cell carcinomas of the colon represent 0.1 to 0.5% of the CRC cases worldwide. The diagnosis is a surprise finding of the histopathology evaluation. Literature supporting this group of colon cancers suggests a correlation between metaplastic colon conditions and subsequent occurrences in adenocarcinomas of the colon. We encountered a primary colon squamous cell carcinoma in a patient with no evidence of any primary outside the colon. Findings unique to the case were rapid progression, presence of micro abscesses and significant weight loss. The patient represented proliferative and possibly metastatic disease and succumbed to it before definitive chemoradiation could be offered. We chose to present this case to add to the existing literature of squamous cell carcinomas of the colon and the fast deterioration before definitive therapy could be given. The work has been reported in line with the SCARE criteria [34].

## 2 Case presentation

**Fig. 1:**
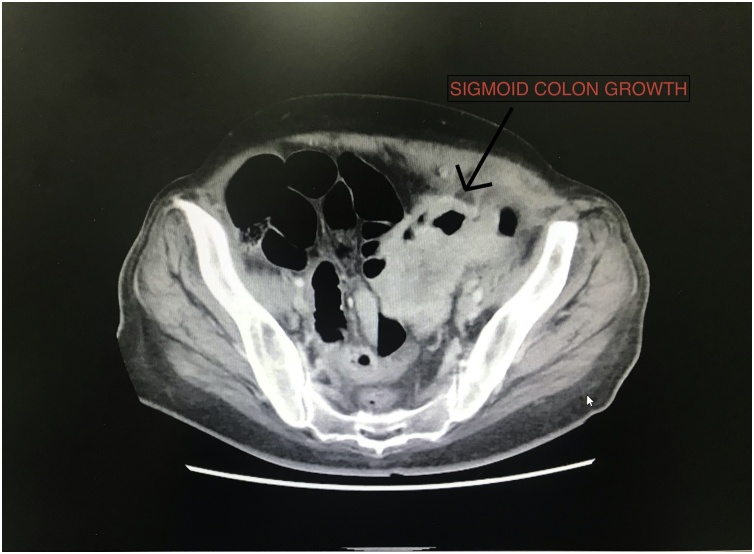
Sigmoid colon growth.

**Fig. 2:**
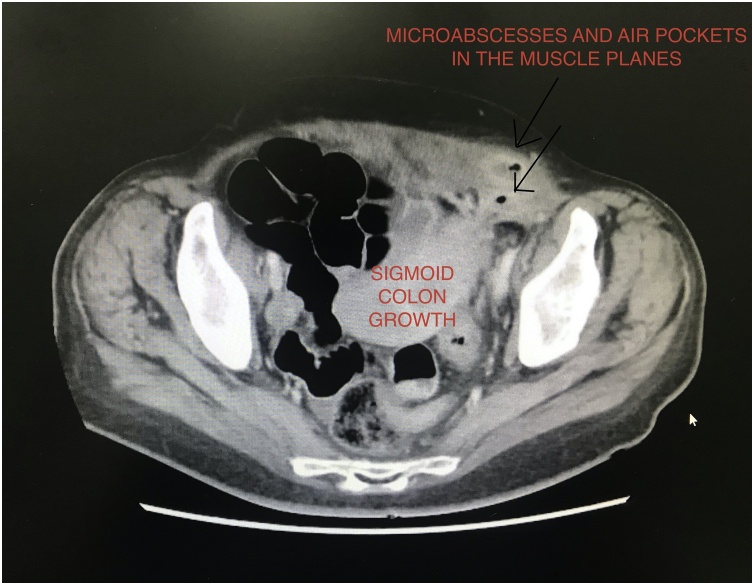
Microabscesses and air pockets in the muscular planes along the sigmoid growth.

A 54-year-old female patient presented to the emergency room with features of intestinal obstruction. She was constipated with evident weight loss over a period of 3 months. She was evaluated by an x-ray abdomen that revealed multiple air fluid levels and the CT – Scan of the abdomen revealed a circumferential growth involving the sigmoid colon with impending perforation and small loculated collections reaching to the superior border of urinary bladder and uterus extending to the lateral abdominal wall suggesting micro abscesses. Gastroscopy performed was normal and the colonoscopy was inconclusive due to narrowing proximal to the sigmoid colon (Figs. 1 and 2).

The patient underwent exploratory laparotomy and sigmoid colectomy subsequently. The findings were an exophytic sigmoid colon tumor proliferating extraluminally with small bowel adhesions. Tumor was adherent to the lateral pelvic abdominal wall with a subcutaneous abscess pocket. Tumor perforation in sigmoid colon was noted with minimal contamination. Tumor was found infiltrating the lateral wall of uterus. No ascites, lymphadenopathy or free peritoneal/omental deposits were noted.

**Fig. 3:**
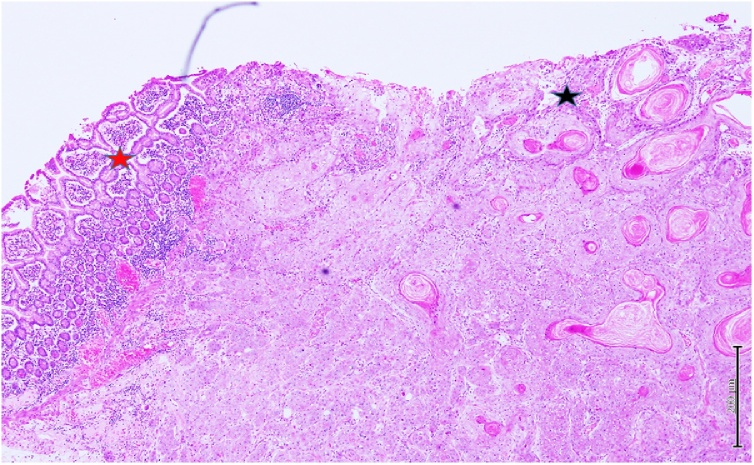
Section showing normal small intestinal mucosa (red star) with adjacent well differentiated keratinizing squamous cell carcinoma in the mucosa extending to deep aspect (black star).

The histopathology report of the patient suggested a moderately differentiated keratinizing squamous cell carcinoma with a single sclerosed lymph node suggesting tumor metastasis with foreign body giant cell reaction (Fig. 3).

The patient underwent extensive evaluation by gynecology and urology departments to identify a possible primary source of malignancy in the urogenital tract. Their evaluations were negative. The patient did not have any skin lesions or ulcers suggesting skin primary as the source.

The patient recovered well from the surgery with parental nutrition support. She was ambulatory and was given oral feeds on 7th postoperative day. Subsequent CT Scan of the abdomen suggested a small fluid collection at the site of surgical anastomosis with few air pockets suggesting abscess formation/leakage. There was concomitant ascites, pleural effusion and a liver lesion noted too in the CT scan. Wound infection was treated with regular dressings initially and later vacuum dressing was applied. After improvement in health, enteral nutrition and wound, the patient was discharged for follow up in 2 weeks. The patient unfortunately passed away at her home and the information was revealed to the surgical team by the patient’s relatives.

## 3 Discussion

Primary colonic squamous cell carcinoma is extremely rare, representing less than 0.5% of all colorectal tumors, with an incidence estimated at 0.1% [1,2]. A look into the research work and the reported cases of SCC dates back to 1907, when Herxheimer reported adenosquamous carcinoma of the cecum but it was in 1919 when the first case of pure SCC of the colon was reported by Schmidtmann [4] in a 65-year-old man [5]. It was not until 1933 that the first case involving the rectum was subsequently described by Raiford [6]. In India, Bhat et al. [7] reported the first case of pure SCC of the colon in 1993 in a 55-year-old female from the southern part of the country.

A look at the available literature reveals that squamous cell carcinoma of the colorectum affects individuals with a mean age of 55–60 years. Women are more frequently predisposed to SCC than men, around 66% of cases occurred in women and 34% in men. SCC colon occurs on average around the fifth decade with a male predominance with increased predilection for the cecum and the right colon [15,16].

The etiopathogenesis of SCC colon is still unclear. It could originate from a multipotent stem cell or develop from squamous metaplasia secondary to chronic irritation [[2], [3], [4],[8], [9], [10]]. In favor of this second hypothesis, the frequent association of SCC colon with chronic inflammatory colitis, in particular ulcerative ulcerative colitis, the relative incidence of this association being 1.7% while it is only 0.25 to 0.5‰ in the general population [5,9]. Several cases have been reported in patients with ulcerative colitis, while others have been in found in association with infections including Schistosomiasis, Entamoeba histolytica and human papilloma virus (HPV) [16,14].

Adenocarcinoma has also been associated with squamous cell cancer of both the colon and rectum. Multiple studies have described either synchronous [11,13] or metachronous lesions [12,13] of adenocarcinoma occurring in the large intestine of patients with squamous cell cancer of the rectum. Additional coexisting diseases have been described including colonic duplication, ovarian cancer, prostate cancer, endometrial cancer, and breast cancer [12].

The clinical characteristics of the patients with SCC of the colorectum are similar to those with adenocarcinoma: rectal bleeding, abdominal pain, change in bowel habits and weight loss [3,17]. Painless hematochezia was seen in few cases of squamous cell and adenosquamous carcinoma of the rectum [18]. Attempts to diagnose and treat early submucosal primary colorectal squamous cell carcinoma (SCC) using endoscopic ultrasound (EUS) technique has not been found as the best diagnostic approach. Surgery remains gold standard with conventional chemoradiation improving efficacy of therapy [32]. Hypercalcemia and persistent hyperleukocytosis were some biochemical abnormalities seen in post-operative course of such patients. Abscess formation may complicate the post-operative course especially when the primary squamous cell carcinoma results in bowel perforation [19].

Before the diagnosis of primary SCC of colorectum is made, certain criteria must be fulfilled as given by Williams et al. in 1979 [23]. This criteria includes: (A) absence of evidence of squamous cell carcinoma of any other part of the body, ruling out any chance of possible metastasis from any organ to the colorectal site; (B) exclusion of any proximal extension of anal squamous cell carcinoma; (C) absence of fistulous tract lined by squamous cells; and (D) confirmation of SCC by histological analysis [20,21,24]. All of these criteria were fulfilled by our case.

Almost four different pathophysiological theories regarding the origin of squamous cell carcinoma of the colorectum have been proposed in the literature which can be summarized as: (A) Proliferation of uncommitted basal cells into squamous cells which undergo malignant transformation following mucosal injury [25]; (B) Ability of pluripotent stem cells to undergo spontaneous squamous differentiation [26]; (C) Squamous metaplasia of glandular epithelium resulting from chronic inflammation or irritation, secondary to inflammatory bowel disease [27], infection [28] or radiation [29]; (D) Origin from embryonal nests of ectodermal cells; and (E) Arousal of carcinomas from preexisting adenomas or adenocarcinomas [22,30].

The Mayo Clinic divided squamous and adenosquamous carcinomas of the colon or rectum into pure squamous – cell carcinoma, mixed adenosquamous carcinoma and adenocarcinoma with benign-appearing squamous metaplasia [31].

The five-year survival after resective therapy for primary squamous-cell and adenosquamous-cell carcinoma of the colon was found to be upto 50 percent for Dukes’ B lesions, 33 percent for Dukes’ C lesions, and 0 percent for Dukes’ D lesions [33].

## 4 Conclusion

Squamous cell carcinoma of the colon can be rarely encountered in CRC histopathology. The surgical treatment remains the same as any CRC and further choice of chemoradiation needs to be addressed. Many patients succumb to the disease rapidly especially if the disease is advanced on presentation and is complicated with micro abscesses and sepsis. Extensive evaluation needs to be done to rule out all sites of possible primary disease before confirming the diagnosis. We recommend further studies into this rare entity to guide further occurrences in the future.

## Declaration of Competing Interest

No conflicts of interest.

## Funding

No funding for the research.

## Ethical approval

Case report approved by the department of general surgery and department of pathology, Bahrain Defence force hospital, Bahrain.

## Consent

Patient and kin consent taken for procedure and the case report publication.

## Author contribution

Dr Aysha Hassan Ali Hussain – First author: Specialist general surgeon involved in the manuscript and treatment of the case.

Dr Kaundinya Kiran B – Second author/corresponding author: Specialist general surgeon involved in the manuscript and treatment of the case.

Dr Faizal Hammed – Third author: Specialist general surgeon involved in the manuscript and treatment of the case.

Dr Abdul Rahim Al Sayed – Fourth author: Consultant general surgeon involved in the treatment of the case and guidance towards manuscript.

## Registration of research studies

1. Name of the registry: N.A.
2. Unique identifying number or registration ID: N.A.
3. Hyperlink to your specific registration (must be publicly accessible and will be checked):

## Guarantor

All authors take full responsibility of the work and the case report.

## Provenance and peer review

Not commissioned, externally peer-reviewed.
